# Jingxin Zhidong Formula Alleviates Tic Disorder via Modulation of Microglial IKK/NF‐κB Signaling and Striatal Neurotransmitter Homeostasis

**DOI:** 10.1002/brb3.71361

**Published:** 2026-03-29

**Authors:** Fei Fan, Jing Yang, Zenghui Niu, Chengkui Xiu, Fei Han

**Affiliations:** ^1^ Department of Paediatrics, Guang'anmen Hospital China Academy of Chinese Medical Sciences Beijing China; ^2^ Experimental Research Center China Academy of Chinese Medical Sciences Beijing China

**Keywords:** GABA, Glutamate, IKK/NF‐κB pathway, Jingxin Zhidong Formula, microglia, neuroinflammation, tic disorder

## Abstract

**Background:**

Tic disorder (TD) is a neurodevelopmental condition for which pharmacological treatments are limited and are frequently associated with side effects. Jingxin Zhidong formula (JXZDF) offers a potential alternative therapy; however, its mechanism of action has not been fully elucidated. This study investigated the therapeutic effects of JXZDF on TD and explored underlying mechanisms involving neurotransmitter regulation and microglial IκB kinase/nuclear factor‐κB (IKK/NF‐κB) signaling, a pathway implicated in both inflammatory activation and metabolic reprogramming.

**Methods:**

A TD rat model was established using 3,3'‐iminodipropionitrile (IDPN). Rats were treated with low‐, medium‐, or high‐dose JXZDF or aripiprazole for three weeks, after which behavioral scores were assessed. Histology, immunohistochemistry, quantitative reverse transcription‐polymerase chain reaction, western blotting (WB), and enzyme‐linked immunosorbent assay (ELISA) analyses of striatal tissue were performed to evaluate neuronal morphology, neurotransmitter receptor expression (N‐methyl‐D‐aspartate receptor subunit 1 [NMDAR1], glutamate receptor 1 [GRIA1], gamma‐aminobutyric acid type A receptor α1 subunit [GABAARα1], and gamma‐aminobutyric acid type A receptor β2 subunit [GABAAβ2]), as well as glutamate and GABA levels. The IKK/NF‐κB pathway was examined both in vivo and in lipopolysaccharide‐stimulated BV‐2 microglial cells using WB, immunofluorescence, and ELISA, with the IKKβ inhibitor IMD‐0354 used for mechanistic validation.

**Results:**

JXZDF treatment significantly reduced abnormal behavioral scores in the TD rat model in a dose‐dependent manner. Treatment ameliorated striatal neuronal damage and corrected the neurotransmitter imbalance by specifically reducing glutamate and increasing the expression of excitatory receptors (NMDAR1, α‐amino‐3‐hydroxy‐5‐methyl‐4‐isoxazolepropionic acid receptors [AMPAR], and glutamate receptor subunit 1 [GluA1]), while increasing GABA and inhibitory receptor expression (GABAARα1 and GABAAβ2). JXZDF also suppressed IKK/NF‐κB pathway activation in the striatum of TD rats and in LPS‐activated BV‐2 cells, as evidenced by reduced IκBα and NF‐κB p65 phosphorylation, inhibited NF‐κB p65 nuclear translocation, and decreased pro‐inflammatory cytokine (interleukin‐6 [IL‐6], tumor necrosis factor‐alpha [TNF‐α]) secretion. The anti‐inflammatory and neuroprotective effects of JXZDF were reversed by co‐treatment with IMD‐0354.

**Conclusions:**

JXZDF alleviated tic‐like behaviors in a TD rat model by restoring striatal excitatory‐inhibitory neurotransmitter balance and inhibiting microglial‐mediated neuroinflammation. Its therapeutic effect was at least partially mediated by suppression of IKK/NF‐κB. These findings provide a pharmacological basis for the clinical application of JXZDF in TD treatment.

## Graphical abstract

Jingxin Zhidong Formula (JXZDF) alleviates tic‐like behaviors in a rat model of tic disorder (TD) by restoring the excitatoryinhibitory neurotransmitter balance in the striatum—reducing glutamate and increasing GABA levels—and by suppressing microglial IKK/NF‐κBmediated neuroinflammation. These effects are dose‐dependent and involve inhibition of NF‐κB nuclear translocation and pro‐inflammatory cytokine release, with mechanistic validation using the IKKβ inhibitor IMD‐0354 in LPS‐stimulated BV‐2 microglia.

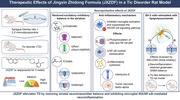

**Abbreviations**
AMPAR: α‐amino‐3‐hydroxy‐5‐methyl‐4‐isoxazolepropionic acid receptor
BSA: bovine serum albumin
BV‐2: murine microglial cell line BV‐2
CNS: central nervous system
CPu: caudate–putamen
ELISA: enzyme‐linked immunosorbent assay
GABA: γ‐aminobutyric acid
GABAARα1: gamma‐aminobutyric acid type A receptor α1 subunit
GABAAβ2: gamma‐aminobutyric acid type A receptor β2 subunit
Glu: glutamate
GluA1: AMPA receptor subunit 1
GRIA1: glutamate receptor subunit 1
HE: hematoxylin and eosin
IDPN: 3,3′‐iminodipropionitrile
IgG: immunoglobulin G
IKK: IκB kinase
IKK/NF‐κB: IκB kinase/nuclear factor‐κB signaling pathway
IL‐6: interleukin‐6
JXZDF: Jingxin Zhidong formula
LPS: lipopolysaccharide
NF‐κB: nuclear factor‐κB
NMDAR1: N‐methyl‐D‐aspartate receptor subunit 1
PBS: phosphate‐buffered saline
qRT‐PCR: quantitative reverse transcription‐polymerase chain reaction
RIPA: radioimmunoprecipitation assay
TBST: Tris‐buffered saline with Tween‐20
TCM: traditional Chinese medicine
TD: tic disorder
TNF‐α: tumor necrosis factor‐alpha
WB: western blotting

## 1 Background

Tic disorder (TD) is a chronic neurodevelopmental disorder characterized by the presence of motor and/or vocal tics, with onset typically occurring in childhood or adolescence. Clinically, TD manifests as uncontrollable, irregular, and involuntary speech or body movements (Deeb et al. 2019; Szejko et al. 2021). Owing to the uniqueness and complexity of TD symptoms, which are often accompanied by mental symptoms such as attention deficit hyperactivity disorder, sleep disorders, obsessive‐compulsive disorder, and functional impairments in multiple domains, TD significantly affects patients’ quality of life (Osland et al. 2018; Essoe et al. 2019; Jalenques et al. 2024). Tics typically emerge in children aged 5 to 6 years, peak in severity between 10 and 12 years of age, and gradually abate by early adulthood (Pringsheim et al. 2019). The prevalence of TD among Chinese children is 6.1%, with a significantly higher incidence in males than in females (approximately 3:1 ratio) (Yang et al. 2016; Wang et al. 2021).

The primary treatments for TD include behavioral interventions, psychotherapy, and pharmacotherapy. Treatment plans must be tailored to the individual needs and goals of patients and their families (Roessner et al. 2022; Woods et al. 2023). Antipsychotic medications represent the most effective pharmacological intervention for TD. α‐2 agonists have demonstrated superior efficacy over placebo and offer an alternative for patients who are reluctant to take antipsychotics (Roessner et al. 2022; Neuchat et al. 2023). However, these medications may have adverse effects, including drowsiness, metabolic syndrome, and extrapyramidal symptoms (Billnitzer and Jankovic 2020). To address these challenges, traditional Chinese medicine (TCM) has emerged as an accepted complementary and alternative therapy for TD and is widely utilized, particularly in China (Pu et al. 2023; Fan et al., 2022).

The pathophysiology of TD is closely linked to dysfunction in the cortico‐striato‐thalamo‐cortical circuit and the dysregulation of neurotransmitter systems within the central nervous system (CNS) (Hall et al. 2025). Glutamate (Glu) and γ‐aminobutyric acid (GABA) are the primary neurotransmitters in the CNS (Kanaan et al. 2016; Hao et al. 2023). Abnormal Glu and GABA concentrations have been observed in the striatum and plasma of patients with TD, although these findings remain controversial (Yu et al. 2023; Chen et al. 2025). Microglia, the innate immune cells of the brain, mediate neuroinflammation by upregulating inflammatory factors and chemokines in the striatum and serum. They also regulate brain development and maintain neuronal homeostasis (Glass et al. 2010; Fleiss et al. 2021). Microglia overactivation can disrupt the oxidative metabolism of medium spiny neurons in the striatum, leading to synaptic dysfunction and contributing to the pathogenesis of various neuropsychiatric disorders, including TD (Kwon and Koh 2020; Wang et al. 2025).

Jingxin Zhidong Formula (JXZDF) is a personalized TCM prescription developed by pediatrician Professor Fei Han. The formula incorporates 13 medicinal herbs: Concha Margaritifera (Zhenzhumu), calcined Os Draconis (Longgu), Concha Ostreae (Muli), Semen Ziziphi Spinosae (Suanzaoren), Semen Platycladi (Baiziren), Radix Paeoniae Alba (Baishao), Bombyx Batryticatus (Jiangcan), Radix Bupleuri (Chaihu), Fructus Arctii (Niubangzi), Fructus Aurantii (Zhiqiao), Lumbricus (Dilong), Periostracum Cicadae (Chantui), and Angelica Dahurica (Baizhi). JXZDF is safe and effective for the treatment of TD (Fan et al. 2022). The mechanism underlying JXZDF in treating TD involves its ability to modulate microglia activation and alter pro‐inflammatory mediator expression (Tian et al. 2023); however, its precise mechanisms of action remain to be fully elucidated. Semen Ziziphi Spinosae ameliorated stereotyped behaviors in a rat model of TD by modulating Glu and GABA levels in the striatal extracellular fluid (Fan et al. 2025). To investigate whether JXZDF modulates Glu and GABA levels in the striatal extracellular fluid during TD, we established in vivo and in vitro models to provide foundational research data supporting its clinical application.

## 2 Methods

### 2.1 Animal Model and Treatments

Eight‐week‐old male Sprague‐Dawley rats (200 to 230 g) were obtained from Liaoning Changsheng Biotechnology Co., Ltd. All animals were housed under controlled conditions (25 ± 1°C, 45%–55% humidity, 12 h light/dark cycle). Rats were group‐housed (3 to 4 animals per cage) in standard polypropylene cages (480 × 350 × 200 mm) with autoclaved wood shavings as bedding. Animals had ad libitum access to sterilized food and water throughout the experiment. All animals were acclimatized to the laboratory conditions for 7 days prior to the start of experiments. A total of 36 rats were randomly assigned to two groups: a control group (*n* = 6) and a TD model induction group (*n* = 30), with no significant body weight difference between the groups (*P* > 0.05). Randomization was performed using a computer‐generated random number table by an investigator not involved in subsequent experiments. The TD model was established as previously described (Diamond et al. 1982). Briefly, 3,3′‐iminodipropionitrile (IDPN) was suspended in 0.9% NaCl (30 mg/mL). Rats received daily intraperitoneal injections of IDPN (250 mg/kg) for seven consecutive days, while control rats received an equal volume of normal saline. Injections were performed between 9:00 a.m. and 11:00 a.m. each day to minimize circadian variation. All endpoints described in this study, including behavioral assessments, histological analyses, qRT‐PCR, western blotting, immunohistochemistry, and ELISA measurements of neurotransmitters and inflammatory cytokines, were pre‐specified prior to data collection. No endpoints were added, removed, or selectively reported after the completion of experiments. All assays were performed on samples from all animals in each group, with no post‐hoc exclusion of data or animals. All procedures were approved by the Ethics Committee of Guang'anmen Hospital, China Academy of Chinese Medical Sciences, and conducted in accordance with the NIH Guide for the Care and Use of Laboratory Animals and the Animal Research: Reporting of In Vivo Experiments guidelines. All animals were monitored daily for general health status, including body weight, food and water intake, grooming behavior, and signs of distress or pain. No significant adverse effects or unexpected mortality was observed during the study. Humane endpoints were predefined as >20% body weight loss over 48 h, inability to reach food or water, or signs of severe pain or distress; however, none of the animals met these criteria during the experiment. At the end of the experiment (day 21 post‐treatment), all rats were euthanized by CO_2_ inhalation in a dedicated chamber with a gradual fill rate (40% of chamber volume per minute), followed by cervical dislocation to ensure death. Death was confirmed by the absence of respiration and lack of pedal reflex. All efforts were made to minimize suffering.

### 2.2 JXZDF Preparation and Treatment

The medicinal components of JXZDF were obtained from the Pharmacy Department of Guang'anmen Hospital, China Academy of Chinese Medical Sciences (Beijing, China). The preparation method is described in detail elsewhere (Tian et al. 2023).

Rats in the control group were administered normal saline for three weeks (10 mL/kg/day; *n* = 6), whereas TD model rats were further randomly divided into five subgroups: the model group (administered normal saline for 3 weeks, 10 mL/kg/day; *n* = 6), the low‐concentration JXZDF group (L‐JXZDF; administered 9.45 g/kg/day of JXZDF for 3 weeks; *n* = 6), the medium‐concentration JXZDF group (M‐JXZDF; administered 18.9 g/kg/day of JXZDF for 3 weeks; *n* = 6), the high‐concentration JXZDF group (H‐JXZDF; administered 37.8 g/kg/day of JXZDF for 3 weeks; *n* = 6), and the aripiprazole group (administered 10 mg/kg/day of aripiprazole for 3 weeks; *n* = 6).

The doses of JXZDF and aripiprazole were selected based on previous research (Chen et al. 2025; Tian et al. 2023). After treatment and behavioral tests, approximately 1.5 mL of blood was collected from the tail vein of each rat into ethylenediaminetetraacetic acid‐coated tubes. Plasma was separated by centrifugation (5000 × g, 10 min, 4°C), aliquoted, and stored at –80°C until further analysis. All rats remained healthy throughout the study, with no unexpected deaths. The procedures complied with institutional ethical and animal welfare guidelines. Striatal tissues were extracted and fixed in 10% neutral buffered formalin for 24 h for pathological examination. The remaining samples were used for quantitative reverse transcription‐polymerase chain reaction (qRT‐PCR), Western blotting, and immunohistochemical analyses.

### 2.3 Behavior Analysis

After a 5‐min acclimation period in the observation cage, each rat underwent a 5‐min double‐blind observation session once every 7 days. Stereotypic behavior was scored according to established criteria from previous research. After each trial, every observation cage was thoroughly cleaned to eliminate any influence on the testing results of the next animal (Al Kadasah et al. 2009).

### 2.4 Hematoxylin and Eosin (HE) Staining

Following fixation in a 4% paraformaldehyde solution, tissue samples underwent dehydration through a graded alcohol series, clearing in xylene, and embedding in paraffin. Sections of 5 µm thickness were obtained and subjected to staining with hematoxylin for approximately 30 min, followed by eosin staining for 1 min. After staining, the sections were rinsed, dehydrated, cleared, and mounted with transparent neutral gum. Tissue morphology was examined using a BX53 optical microscope (OLYMPUS). Images were acquired using a DP73 microscope photography system (OLYMPUS).

### 2.5 Nissl Staining

Striatum tissue from rats in each group was paraffin‐embedded and stained with cresyl violet (Nissl stain). The sections were first dehydrated, and each slide was covered with a 0.5% cresyl violet solution and stained at room temperature for 10 min. Subsequently, the sections were briefly differentiated in a 0.25% acetic acid‐ethanol solution, followed by dehydration, clearing, and mounting. The samples were visualized using a BX53 optical microscope (OLYMPUS), and images were captured using the DP73 microscope photography system (OLYMPUS).

### 2.6 Immunohistochemistry

Following deparaffinization and antigen retrieval, paraffin‐embedded rat striatum sections were incubated with primary antibodies (1:100 dilution) overnight at 4°C. Subsequently, the sections were incubated with a biotin‐labeled secondary antibody (1:400) at 37°C for 60 min, followed by three 5‐min washes in phosphate‐buffered saline (PBS). 3,3'‐diaminobenzidine chromogen was applied for color development. The sections were subsequently rinsed, counterstained, dehydrated, cleared, and mounted with neutral gum. For semi‐quantitative analysis, three random fields per section were captured at 800× magnification using Image‐Pro Plus 6.0 software (Media Cybernetics, Inc., Rockville, MD, USA). The integrated optical density and area of positive regions were measured, and their ratio was calculated. The primary and secondary antibodies used for immunohistochemistry were as follows: anti‐NMDAR1 (ALS18624, Abcepta, China), anti‐AMPAR GluA1 (R381355, Zenbio, China), anti‐GABAARα1 (161697, Zenbio, China), anti‐GABAAβ2 (AP60868, Abcepta, China), anti‐IKKα (WL00053, Wanleibio, China), anti‐IKKβ (WL04340, Wanleibio, China), anti‐IκBα (WL01936, Wanleibio, China), anti‐p‐IκBα (S32) (WLH3930, Wanleibio, China), anti‐NF‐κB p65 (WL01980, Wanleibio, China), and anti‐p‐NF‐κB p65 (S536) (310013, Zenbio, China). The biotin‐labeled secondary antibody was an HRP‐conjugated goat anti‐rabbit IgG (#31460, ThermoFisher, USA).

### 2.7 Cell Culture and Lipopolysaccharide (LPS) Treatment

The BV‐2 microglial cell line was obtained from the Cell Resource Center of the Institute of Basic Medical Sciences, Chinese Academy of Medical Sciences. Cells were cultured in Dulbecco's Modified Eagle Medium supplemented with 10% fetal bovine serum and 100 U/mL penicillin‐streptomycin and maintained at 37°C in a humidified 5% CO_2_ incubator. For experiments, cells in logarithmic‐phase growth were seeded into 6‐well plates at 1 × 10^4^ cells/mL. At approximately 80% confluence, the cells were stimulated with 1 µg/mL LPS for 24 h to establish an activated BV‐2 model (Tian et al. 2023). The cells were then divided into six groups: control (untreated), LPS (1 µg/mL LPS only), LPS+L‐JXZDF (1 µg/mL LPS + 12.5 mg/mL JXZDF), LPS+M‐JXZDF (1 µg/mL LPS+25 mg/mL JXZDF), LPS+H‐JXZDF (1 µg/mL LPS + 50 mg/mL JXZDF), and aripiprazole (1 µg/mL LPS+8.97 µg/mL aripiprazole). The drug doses were based on previous studies (Tian et al., 2023; Racki et al., 2021). To further examine the role of the IκB kinase/nuclear factor‐κB (IKK/NF‐κB) pathway in the effects of JXZDF, the IKKβ inhibitor IMD‐0354 (10 ng/mL) was applied to the H‐JXZDF and aripiprazole groups for additional validation, as described previously (Hikage et al. 2021).

### 2.8 qRT‐PCR

Total RNA was extracted from rat striatal tissue using TRIzol reagent (BioTeke Corporation, Beijing, China) and quantified using a NanoDrop One UV spectrophotometer (Thermo Fisher Scientific, Waltham, MA, USA). RNA was reverse‐transcribed to cDNA using All‐in‐One First‐Strand SuperMix (Guangzhou Meiji Biotechnology Co., Ltd.). PCR amplification was carried out using 2× Fast Taq Plus PCR Master Mix (Biosharp) on a Pangaea 3 fluorescence quantitative PCR instrument (Aperbio). Relative gene expression was calculated using the 2−ΔΔCt method, with β‐actin serving as the endogenous control. The primer sequences are provided in Table 1.

**TABLE 1:** Primer information.

Primer name	Sequence
NMDAR1 forward	5′‐TCTACGCAACTGTAAAGCA‐3′
NMDAR1 reverse	5′‐GCCGAGTCCCAGATAAA‐3′
Gria1 forward	5′‐CCATCCGTGTTTGTTCG‐3′
Gria1 reverse	5′‐CAGGGCTTTCGTTGCTC‐3′
GABRAɑ1 forward	5′‐AGAGGGTATGCGTGGGATG‐3′
GABRAɑ1 reverse	5′‐CCAAATAGCAGCGGAAAGG‐3′
GABRBβ2 forward	5′‐AATAATCGCCGCTGTCT‐3′
GABRBβ2 reverse	5′‐TCCGAAATCTGGTCTCA‐3′
β‐actin forward	5′‐TGTCACCAACTGGGACGATA‐3′
β‐actin reverse	5′‐GGGGTGTTGAAGGTCTCAAA‐3′

Abbreviations: GABRAα1, gamma‐aminobutyric acid type A receptor α1 subunit; GABRBβ2, gamma‐aminobutyric acid type A receptor β2 subunit; Gria1, glutamate receptor subunit 1; NMDAR1, N‐methyl‐D‐aspartate receptor subunit 1.

### 2.9 Western Blotting

For both rat striatal tissues and cultured BV‐2 cells, total proteins were extracted using ice‐cold radioimmunoprecipitation assay (RIPA) lysis buffer, supplemented with protease and phosphatase inhibitors. For tissue samples, approximately 30 mg of striatum was homogenized and centrifuged at 12,000 × g for 15 min at 4°C. For cell samples, after PBS washes, pellets were lysed in RIPA buffer, incubated on ice for 30 min, and centrifuged at 12,000 × g for 10 min at 4°C. The supernatants were collected and stored at −80°C until further analysis.

For immunoblotting, 30 µg of protein per lane was separated by sodium dodecyl sulfate‐polyacrylamide gel electrophoresis (80 V in stacking gel, 120 V in separating gel) and transferred onto a polyvinylidene difluoride membrane using a semi‐dry system. Following blocking with 5% non‐fat milk in Tris‐buffered saline with Tween‐20 (TBST) for 1 h at room temperature, the membrane was incubated overnight at 4°C with primary antibodies diluted in bovine serum albumin (BSA)/TBST. After washing three times with TBST (10 min each), the membrane was incubated with an HRP‐conjugated secondary antibody for 2 h at room temperature. Finally, the protein bands were visualized with enhanced chemiluminescence and imaged using a chemiluminescence system. Grayscale values were analyzed using ImageJ software (National Institutes of Health, USA). The primary antibodies used for Western blotting were as follows: anti‐NMDAR1 (ALS18624, Abcepta, China), anti‐AMPAR GluA1 (R381355, Zenbio, China), anti‐GABAARα1 (161697, Zenbio, China), anti‐GABAAβ2 (AP60868, Abcepta, China), anti‐IKKα (WL00053, Wanleibio, China), anti‐IKKβ (WL04340, Wanleibio, China), anti‐IκBα (WL01936, Wanleibio, China), anti‐p‐IκBα (Ser32) (WLH3930, Wanleibio, China), anti‐NF‐κB p65 (WL01980, Wanleibio, China), and anti‐p‐NF‐κB p65 (Ser536) (WL02169, Wanleibio, China). The horseradish peroxidase‐conjugated secondary antibody was goat anti‐rabbit IgG‐HRP (WLA023, Wanleibio, China). The internal control antibody was anti‐β‐actin (WL01372, Wanleibio, China).

### 2.10 Enzyme‐linked Immunosorbent Assay (ELISA)

Striatal tissue samples were weighed and homogenized in ice‐cold PBS at a 1:9 (w/v) ratio. Following centrifugation at 2795 × g for 5 min at 4°C, the supernatant was collected for analysis. Glu and GABA concentrations were quantified using specific commercial ELISA kits (Dopamine ELISA Kit, EU0392, Wuhan Fein Biotechnology Co., Ltd., China; BCA Protein Concentration Assay Kit, WLA004, Wanleibio, China) according to the manufacturer's protocols. Absorbance was measured at 450 nm with a correction at 570 nm using an ELX‐800 microplate reader (BioTek Instruments, Inc., USA). Sample concentrations were determined by interpolating the corrected optical density values against a standard curve.

### 2.11 Immunofluorescent Staining

BV‐2 cells were seeded in black 96‐well plates at a density of 1 × 10^4^ cells per well. After overnight incubation to allow for attachment, they were treated with the indicated drugs for a period of 24 h. After treatment, the cells were washed twice with PBS, fixed using 4% paraformaldehyde (100 µL/well, 15 min at room temperature), and subsequently washed three times with PBS (5 min each). Following fixation, cells were permeabilized with 0.5% Triton X‐100 (100 µL/well for 5 min at room temperature) and then washed three times with PBS. After blocking with 1% BSA (1 h at room temperature), the cells were incubated with rabbit anti‐NF‐κB primary antibody (NF‐κB p65 Recombinant Rabbit mAb, Bioss, bs‐m‐52305R, 1:100, 1.5  h at room temperature), washed, and then incubated with 594‐conjugated goat anti‐rabbit immunoglobulin G (IgG) secondary antibody (Multi‐rAb CoraLite Plus 594‐Goat Anti‐Rabbit Recombinant Secondary Antibody, Proteintech, RGAR004, 1:300, 1 h in the dark). Following the final PBS washes, images were acquired using a fluorescence microscope (IX83; OLYMPUS) at 100× magnification. For quantification, five randomly selected fields per well were captured from three independent experimental replicates. Sampling was performed by an investigator blinded to group allocation, using a systematic random sampling method to avoid bias. Nuclear and cytoplasmic fluorescence intensities of NF‐κB p65 were measured separately using ImageJ software (National Institutes of Health, USA).

### 2.12 Statistical Analysis

GraphPad Prism (version 8.0) was employed for statistical data analysis (GraphPad Software, Boston, MA, USA). Data are presented as means ± standard deviation. Statistical significance across multiple groups was determined using one‐way ANOVA with Tukey's post hoc correction. Behavioral scores, expressed as medians (interquartile range), were analyzed using the Kruskal–Wallis test followed by Dunn's post hoc test for correction. Statistical significance was set at *P* < 0.05. To minimize analytical flexibility and ensure transparency, all statistical tests were pre‐defined, and no post‐hoc selection of endpoints or subgroups was performed. For all in vivo experiments, *n* represents the number of animals per group, and each animal was considered an independent biological replicate. For in vitro experiments, n represents the number of independent experimental replicates, each performed in triplicate.

## 3 Results

### 3.1 Behavior Analysis

Rat behavior was assessed on days  7 (post‐modeling) and 21 (post‐treatment). The model group showed significantly higher scores (>2) than the control group,  confirming successful TD modeling. Following treatment, all JXZDF‐treated groups (L‐, M‐, and H‐JXZDF) exhibited significantly lower scores than the model group (Table 2).

**TABLE 2:** Behavior scores according to treatment group (median [Q1, Q3]) (unit: Points).

Group	Post‐induction	Post‐treatment (Day 21)
A	0	0
B	4.00 (3.00, 4.00)	4.00 (3.00, 4.00)
C	3.00 (3.00, 4.00)	1.00 (1.00, 2.00)^*#^
D	3.50 (3.00, 4.00)	1.50 (1.00, 2.00)^*#^
E	4.00 (3.00, 4.00)	1.00 (1.00, 1.00)^*#^
F	4.00 (3.00, 4.00)	1.00 (1.00, 1.00)^*#^

*Note*: Behavioral scores were analyzed using the Kruskal–Wallis test and Dunn's post hoc test. ^*^ *P* < 0.05 vs. the same group post‐modeling; ^#^ *P* < 0.05 vs. the model group at the same time point. Group codes: A, Control; B, Model; C, low‐dose JXZDF; D, medium‐dose JXZDF; E, high‐dose JXZDF; F, aripiprazole.

### 3.2 Effects of JXZDF on Neuronal Morphology in the Rat Striatum

**FIGURE 1:**
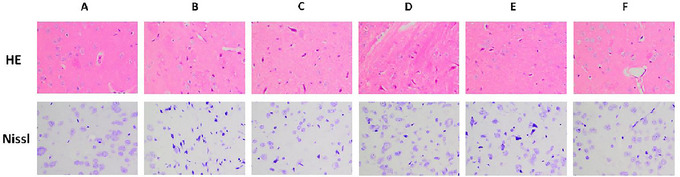
Representative hematoxylin‐eosin and Nissl staining images of rat striatum tissue (800× magnification). Group codes: A, Control; B, Model; C, low‐dose JXZDF; D, medium‐dose JXZDF; E, high‐dose JXZDF; F, aripiprazole. Abbreviation: HE, hematoxylin and eosin.

Histopathological analysis of the rat striatum using HE and Nissl staining revealed significant neuronal damage in the model group, including shrunken neurons, cytoplasmic vacuolation, and reduced Nissl body density. Treatment with JXZDF ameliorated these injuries in a dose‐dependent manner, and the high‐dose group showed nearly normal morphology and preserved Nissl bodies. Aripiprazole exhibited strong neuroprotective effects, maintaining striatal structure and Nissl body integrity similar to those observed in the controls (Figure 1).

### 3.3 JXZDF Modulates Glu and GABA Receptor Expression in the Rat Model of TD

**FIGURE 2:**
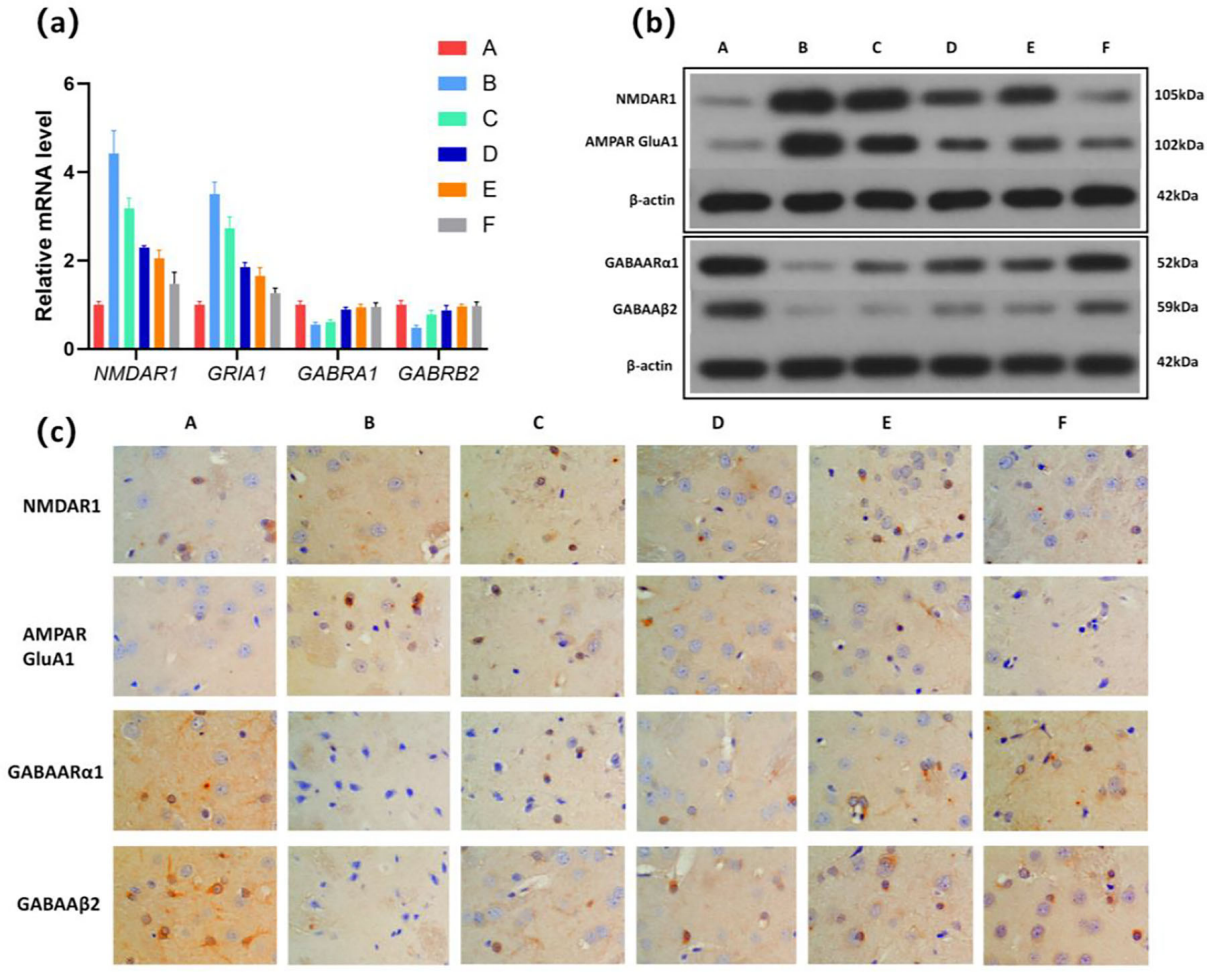
JXZDF modulates glutamate levels and GABA receptor expression in the rat model of TD**. (a)** qRT‐PCR results showing mRNA expression levels of *NMDAR*1, *GRIA1*, *GABAARα1*, and *GABAAβ2*, **(b)**Western blot results showing the protein levels of NMDAR1, AMPAR GluA1, GABAARα1, and GABAAβ2, and **(c)** Immunohistochemistry results show the protein levels of NMDAR1, AMPAR, GluA1, GABAARα1, and GABAAβ2 (800× magnification). Group codes: A, Control; B, Model; C, low‐dose JXZDF; D, medium‐dose JXZDF; E, high‐dose JXZDF; F, aripiprazole. Abbreviations: AMPAR, α‐amino‐3‐hydroxy‐5‐methyl‐4‐isoxazolepropionic acid receptor; GABA, γ‐aminobutyric acid; GABAARα1, gamma‐aminobutyric acid type A receptor α1 subunit; GABAAβ2, gamma‐aminobutyric acid type A receptor β2 subunit; GluA1, AMPA receptor subunit 1; GRIA1, glutamate receptor subunit 1; JXZDF, Jingxin Zhidong formula; NMDAR1, N‐methyl‐D‐aspartate receptor subunit 1.

qRT‐PCR analysis demonstrated that compared with controls, JXZDF significantly reversed the dysregulated expression of key neurotransmitter receptor subunits in the striatum of TD model rats, while the model group showed marked downregulation of GABAA receptor subunits (GABRA1 and GABRB2) and upregulation of ionotropic glutamate receptor subunits (GRIA1 and NMDAR1) (*P* < 0.01). JXZDF administration dose‐dependently restored these mRNA levels, with high‐dose JXZDF reaching levels comparable to those in the control group. This effect was also observed in the aripiprazole group (Figure 2a). Consistent with these findings, immunohistochemistry revealed increased NMDAR1 and AMPAR GluA1 immunoreactivity and decreased GABAARα1 and GABAAβ2 staining in the model group, which were dose‐dependently reversed by JXZDF treatment (Figure  2c, Table  3).

**TABLE 3:** Neurotransmitter receptor subunit expression levels in rat CPu tissue according to treatment group ($\bar{X}$± s).

Group	Number	NMDAR1	AMPAR GluA1	GABAARα1	GABAAβ2
A	3	0.0043 ± 0.0012	0.0005 ± 0.0001	0.0450±0.0125	0.0415±0.0094
B	3	0.0244 ± 0.0064^*^	0.0050 ± 0.0007^*^	0.0011 ± 0.0002^*^	0.0044 ± 0.0008^*^
C	3	0.0148 ± 0.0039^*#^	0.0035 ± 0.0008^*#^	0.0016 ± 0.0002^*#^	0.0084 ± 0.0015^*#^
D	3	0.0114 ± 0.0025^*#^	0.0028 ± 0.0004^*#^	0.0175 ± 0.0046^*#^	0.0153 ± 0.0043^*#^
E	3	0.0107 ± 0.0021^*#^	0.0014 ± 0.0002^*#^	0.0203 ± 0.0029^*#^	0.0187 ± 0.0046^*#^
F	3	0.0079 ± 0.0021^*#^	0.0007 ± 0.0001^*#^	0.0281 ± 0.0044^*#^	0.0277 ± 0.0057^*#^

*Note*: The model group was compared with the control group. ^*^ *P* <0.05 compared with the model group at the same time point, ^#^ *P* < 0.05. Group codes: A, Control; B, Model; C, low‐dose JXZDF; D, medium‐dose JXZDF; E, high‐dose JXZDF; F, aripiprazole.

Abbreviations: AMPAR, α‐amino‐3‐hydroxy‐5‐methyl‐4‐isoxazolepropionic acid receptor; CPu, caudate‐putamen; GABAARα1, gamma‐aminobutyric acid type A receptor α1 subunit; GABAAβ2, gamma‐aminobutyric acid type A receptor β2 subunit; GluA1, AMPA receptor subunit 1; NMDAR1, N‐methyl‐D‐aspartate receptor subunit 1;.

Western blot analysis further confirmed that JXZDF attenuated the upregulated expression of excitatory receptors (NMDAR1 and AMPAR GluA1) and restored the downregulated expression of inhibitory receptors (GABAARα1 and GABAAβ2) in the striatum (Figure 2b). This restorative effect was most evident in the M‐JXZDF, H‐JXZDF, and aripiprazole groups, indicating that JXZDF ameliorated the excitatory‐inhibitory imbalance in TD by concurrently suppressing excessive glutamate receptor expression and enhancing deficient GABA receptor expression.

### 3.4 JXZDF Modulates Neurotransmitter Levels in the Striatum of TD Rats

**FIGURE 3:**
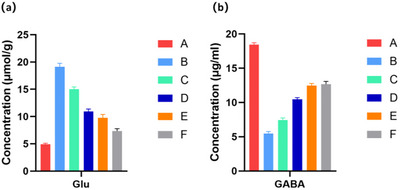
JXZDF modulates Glu and GABA levels in the striatum of TD rats. ELISA results showing Glu **(a)** and GABA **(b)** levels in the striatum of TD model rats. Group codes: A, Control; B, Model; C, low‐dose JXZDF; D, medium‐dose JXZDF; E, high‐dose JXZDF; F, aripiprazole. Abbreviations: GABA, γ‐aminobutyric acid; Glu, glutamate; JXZDF, Jingxin Zhidong formula; TD, tic disorder.

ELISA measurements of striatal levels of Glu and GABA in each experimental group revealed that compared with controls, the model group showed significantly increased Glu and decreased GABA levels. JXZDF treatment, especially at medium and high doses, substantially attenuated the Glu elevation and rescued the GABA deficit, with the high‐dose group showing GABA levels comparable to those in the aripiprazole group (Figure 3).

In summary, these results showed that JXZDF rebalanced the striatal neurotransmitter profile in the rat model of TD by lowering excess Glu and raising deficient GABA levels.

### 3.5 JXZDF Inhibits the IKK/NF‐κB Pathway in TD Rats

**FIGURE 4:**
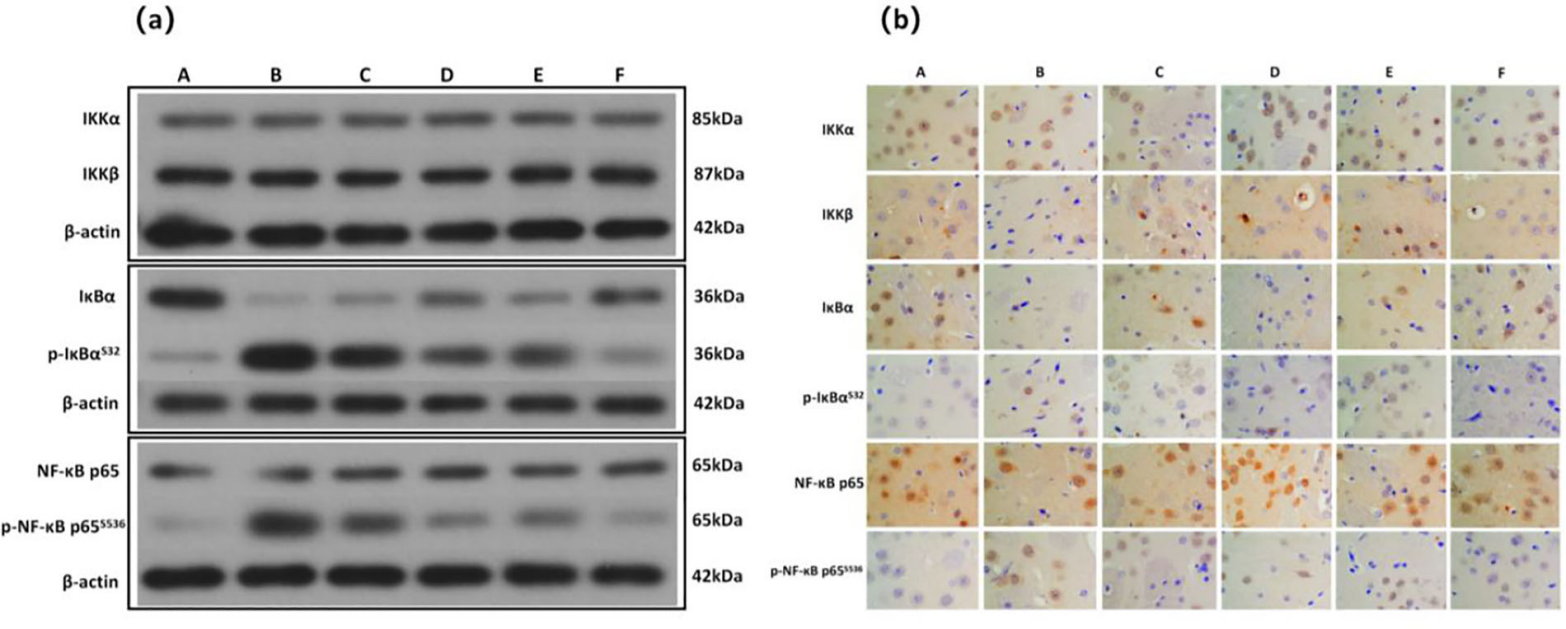
JXZDF inhibits the IKK/NF‐κB pathway in the rat model of TD**. (a)** Western blot showing the protein levels of IKKα, IKKβ, IκBα,p‐IκBα, NF‐κB p65, and p‐NF‐κB p65 and **(b)** Immunohistochemistry results showing the protein levels of IKKα, IKKβ, IκBα, p‐IκBα, NF‐κB p65, and p‐NF‐κB p65 (800× magnification). Group codes: A, Control; B, Model; C, low‐dose JXZDF; D, medium‐dose JXZDF; E, high‐dose JXZDF; F, aripiprazole. Abbrevitations: IKK/NF‐κB, IκB kinase/nuclear factor‐κB; JXZDF, Jingxin Zhidong formula; TD, tic disorder.

To further investigate the molecular mechanisms underlying the therapeutic effects of JXZDF, we examined its impact on the IKK/NF‐κB signaling pathway, a key regulator of inflammation and immune response (Kondylis et al. 2017). Western blot analysis of striatal tissue from TD model rats revealed significant upregulation of p‐IκBα and p‐NF‐κB p65 compared with controls, indicating pathway activation (Figure 4a). JXZDF treatment dose‐dependently reversed these changes, with the high‐dose group showing levels closest to those observed in the controls.

Consistent with these findings, the immunohistochemistry results revealed markedly increased staining for IκBα, p‐IκBα, and p‐NF‐κB p65 in the model group. These levels were substantially reduced following JXZDF or aripiprazole treatment in a dose‐dependent manner (Figure  4b, Table 4).

**TABLE 4:** Protein expression levels in rat CPu tissue according to treatment group ($\bar{X}$± s).

Group	Number	IKKα	IKKβ	IκBα	p‐IκBα	NF‐κB p65	p‐NF‐κB p65
A	3	0.0058 ± 0.0016	0.0238 ± 0.0045	0.0177 ± 0.0051	0.0011 ± 0.0003	0.0124 ± 0.0020	0.0038 ± 0.0008
B	3	0.0056 ± 0.0009	0.0231 ± 0.0031	0.0019 ± 0.0003^*^	0.0125 ± 0.0031^*^	0.0110 ± 0.0018	0.0139 ± 0.0023^*^
C	3	0.0057 ± 0.0011	0.0235 ± 0.0004	0.0077 ± 0.0021^*#^	0.0085 ± 0.0016^*#^	0.0108 ± 0.0032	0.0117 ± 0.0027^*^
D	3	0.0059 ± 0.0012	0.0227 ± 0.0023	0.0084 ± 0.0025^*#^	0.0066 ± 0.0014^*#^	0.0121 ± 0.0032	0.0089 ± 0.0017^*#^
E	3	0.0058 ± 0.0017	0.0209 ± 0.0029	0.0090 ± 0.0025^*#^	0.0054 ± 0.0015^*#^	0.0111 ± 0.0020	0.0084 ± 0.0024^*#^
F	3	0.0055 ± 0.0010	0.0215 ± 0.0008	0.0113 ± 0.0026^#^	0.0038 ± 0.0010^*#^	0.0103 ± 0.0030	0.0070 ± 0.0020^*#^

*Note*: The model group was compared with the control group, ^*^ *P* <0.05; compared with the model group at the same time point, ^#^ *P* < 0.05. Group codes: A, Control; B, Model; C, low‐dose JXZDF; D, medium‐dose JXZDF; E, high‐dose JXZDF; F, aripiprazole; IKK, IκB kinase; CPu, caudate‐putamen.

These results indicated that JXZDF alleviates TD by inhibiting the IKK/NF‐κB signaling pathway.

### 3.6 BV‐2 Cell Activation

**FIGURE 5:**
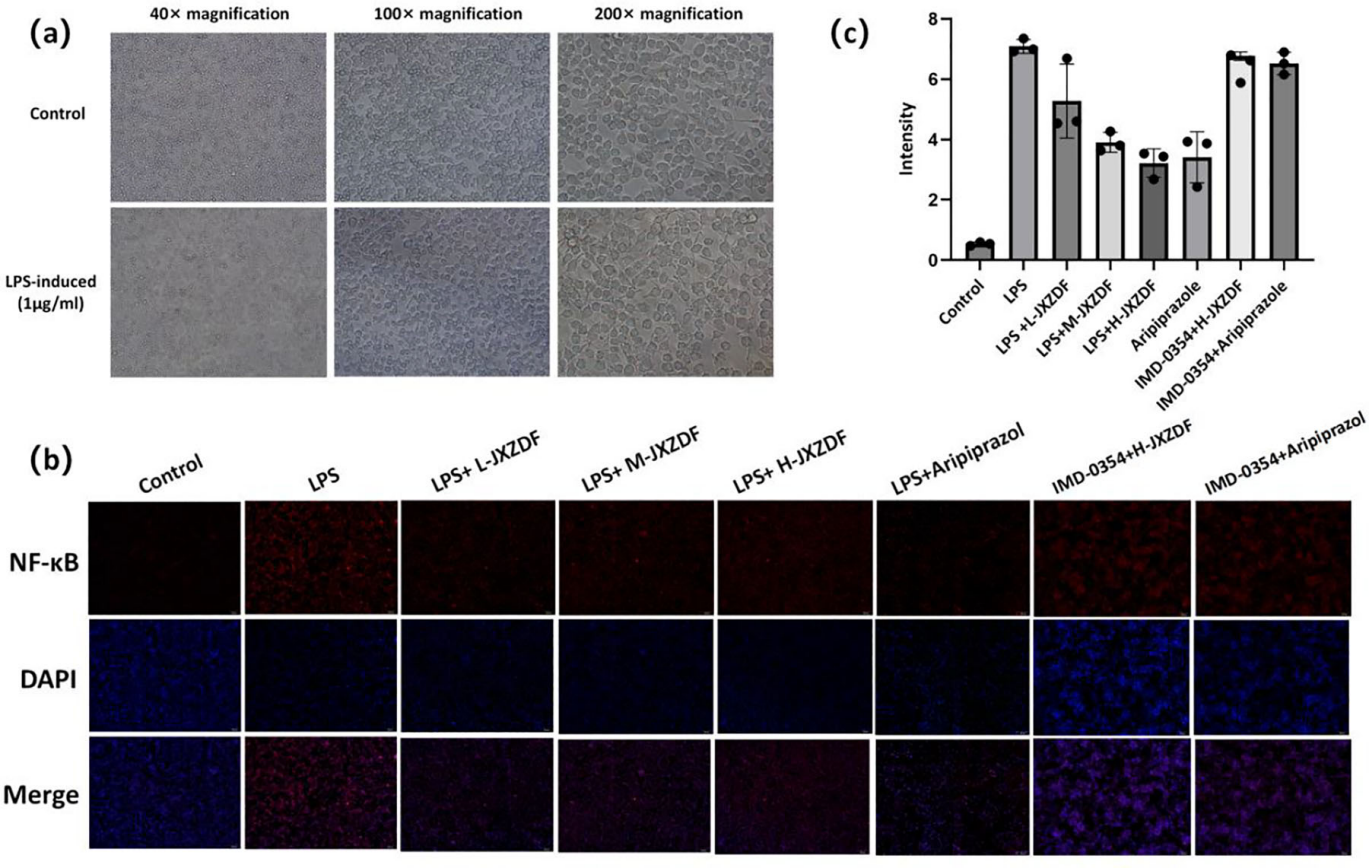
Effects of JXZDF on LPS‐activated BV‐2 microglial cells. **(a)** Morphological changes of BV‐2 cells, **(b)** Immunofluorescence staining showing NF‐κB p65 subcellular localization in BV‐2 cells, and **(c)** Semi‐quantitative analysis of nuclear‐to‐cytoplasmic fluorescence intensity ratios of NF‐κB p65. Abbreviations: JXZDF, Jingxin Zhidong formula; LPS, lipopolysaccharide; NF‐κB p65, p65 (RelA) subunit of the nuclear factor‐κB transcription complex.

After 24 h of exposure to 1 µg/mL LPS, BV‐2 cell morphology was examined. As shown in Figure 5a, LPS treatment markedly altered the cellular morphology compared with the normal control group. Activated BV‐2 cells exhibited enlarged, rounded, or oval cell bodies, with shortened or completely retracted processes, thus displaying a characteristic amoeboid shape (Dirscherl et al. 2010).

### 3.7 JXZDF Inhibits the IKK/NF‐κB Pathway in BV‐2 Cells

We performed immunofluorescence staining for NF‐κB p65 in LPS‐stimulated BV‐2 microglia to evaluate the cellular effects of JXZDF on the IKK/NF‐κB pathway. In control cells, NF‐κB p65 was mainly localized in the cytoplasm, whereas LPS stimulation induced prominent nuclear translocation, indicating pathway activation. JXZDF treatment dose‐dependently suppressed this nuclear translocation, with the high‐dose group showing cytoplasmic retention similar to that observed in the controls (Figure 5b). Semi‐quantitative analysis of nuclear‐to‐cytoplasmic fluorescence ratios further confirmed a significant increase in the LPS group and a dose‐dependent decrease following JXZDF treatment (Figure 5c). To verify pathway specificity, we performed co‐treatment by administering the IKKβ inhibitor IMD‐0354 (10 ng/mL) to the H‐JXZDF and aripiprazole groups. This co‐treatment reversed the inhibitory effect of both drugs, restoring NF‐κB p65 nuclear localization to levels comparable to those in the LPS‐only group.

These results confirmed that JXZDF suppresses NF‐κB activation specifically through the IKK/NF‐κB signaling cascade in microglia.

**FIGURE 6:**
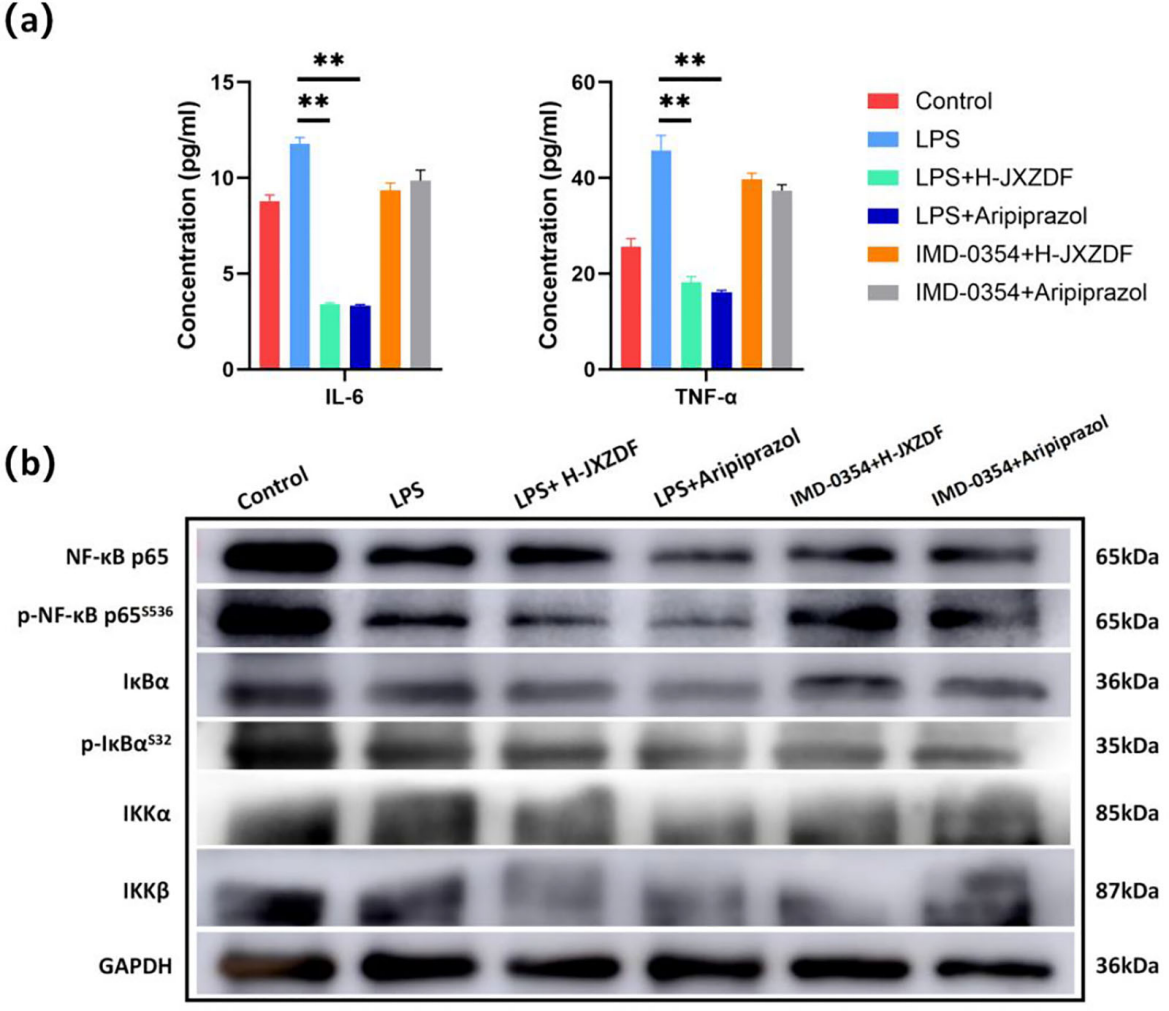
JXZDF inhibits the IKK/NF‐κB pathway in LPS‐stimulated BV‐2 cells via an IKKβ‐dependent mechanism. **(a)** ELISA results showing IL‐6 and TNF‐α levels in the culture supernatants of BV‐2 cells.**P < 0.01 vs. the LPS group; **(b)** Western blot analysis of protein expression in BV‐2 cells. Abbreviations: ELISA, enzyme‐linked immunosorbent assay; IKK/NF‐κB, IκB kinase/nuclear factor‐κB; IL‐6, interleukin‐6; LPS, lipopolysaccharide; TNF‐α, tumor necrosis factor‐alpha; XZDF, Jingxin Zhidong formula.

ELISA results (Figure 6a) demonstrated that LPS stimulation dramatically increased the secretion of the pro‐inflammatory cytokines IL‐6 and TNF‐α, which was significantly reduced by H‐JXZDF and aripiprazole treatments. However, treatment with IMD‐0354 reversed these cytokine‐suppressive effects, restoring IL‐6 and TNF‐α levels close to those observed in the LPS‐stimulated group.

Western blot analysis revealed that LPS stimulation increased the phosphorylation levels in all treatment groups relative to the control group, confirming robust activation of the pathway (Figure 6b).

Treatment with H‐JXZDF or aripiprazole significantly attenuated these LPS‐induced phosphorylation events, restoring the levels of all indicators to baseline. Notably, the total IκBα protein level decreased after LPS stimulation, while the levels of its phosphorylated form (p‐IκBα) significantly increased. This opposing trend is characteristic of canonical IKK/NF‐κB pathway activation. However, the co‐administration of H‐JXZDF or aripiprazole with IMD‐0354 largely abolished the suppressive effects on NF‐κB p65 phosphorylation. The phosphorylation levels in the combination groups returned to values comparable to those in the LPS‐only group, indicating that JXZDF‐ and aripiprazole‐induced inhibition is mediated primarily through the IKKβ‐dependent arm of the NF‐κB signaling cascade.

These results demonstrated that JXZDF suppresses microglial inflammatory activation by specifically targeting the IKK/NF‐κB pathway and that this inhibitory effect depends on functional IKKβ activity.

## 4 Discussion

TCM theory generally categorizes TD as a “wind syndrome,” characterized by symptoms including muscle twitches, tremors, itching, numbness, and spasms (Wang et al. 2025). JXZDF is designed to nourish the heart; calm the spirit and liver; and suppress pathogenic wind. The principal herbs include calcined Os Draconis (Longgu), calcined Concha Ostreae (Muli), and Concha Margaritifera (Zhenzhumu), while Semen Ziziphi Spinosae (Suanzaoren) and Semen Platycladi (Baiziren) are adjuvant herbs (Fan et al. 2022). The combination of these herbs produces therapeutic effects by calming the spirit and suppressing liver wind (Yu et al. 2025). A randomized clinical trial demonstrated the treatment safety, with stable and significant clinical effectiveness, suggesting that it warrants further investigation (Fan et al. 2022).

In the present study, JXZDF treatment significantly reduced behavioral scores in a rat model of TD, indicating its efficacy in alleviating tic symptoms. JXZDF was also associated with distinct pathological changes in the brain, including damage to Nissl bodies in the striatum, increased neuronal count in TD rats, mitigated brain tissue damage, and reduced injury to striatal Nissl bodies, further highlighting its therapeutic potential.

Imbalanced glutamatergic expression in the brain contributes to TD. Elevated Glu levels in patients with TD are associated with neuronal hyperexcitability, whereas reducing brain Glu levels significantly ameliorates tic behavior in animal models (Hao et al. 2023). GABA is a major inhibitory neurotransmitter widely distributed in the CNS (Jurgen and Greenberg 2024). By binding to GABA receptors on postsynaptic neurons, GABA modulates ion channels, promotes membrane hyperpolarization, reduces neuronal excitability, and helps regulate emotion and excitatory behaviors. Reduced GABA concentrations in the primary sensorimotor cortex, supplementary motor area, and insular cortex are significantly associated with greater severity and frequency of premonitory urges (He et al. 2021). The results demonstrated significant alterations in excitatory/inhibitory balance in the TD model, characterized by increased glutamate and decreased GABA levels relative to control animals. Concurrently, behavioral scores differed significantly in TD rats, suggesting that changes in Glu and GABA may be linked to behavioral manifestations in TD. JXZDF treatment reversed the aberrant levels of Glu and GABA and returned the behavioral scores of TD rats to levels comparable to those in healthy controls. Thus, JXZDF may exert therapeutic effects in the TD rat model by reducing glutamate accumulation and enhancing GABAergic expression. Finally, the therapeutic effect of JXZDF was dose‐dependent, with the high‐dose treatment group showing the most pronounced results.

We also explored the molecular mechanisms underlying the therapeutic effects of JXZDF. The NF‐κB signaling pathway is a central regulator of a broad spectrum of genes that mediate immune and inflammatory responses (Tian et al. 2023). The canonical NF‐κB pathway is activated by the IKK complex (Lim et al. 2017). Upon receiving specific stimuli, IKK is activated, leading to NF‐κB dissociation from its inhibitory complex, its translocation to the nucleus, and the initiation of gene transcription, which affects the expression of various cytokines. In microglia, the IKK/NF‐κB signaling pathway is essential for the expression of pro‐inflammatory genes associated with the M1 phenotype (Del Moro et al. 2025). Therefore, we hypothesized that JXZDF's modulation of the IKK/NF‐κB pathway might be a molecular mechanism mitigating disease progression. We observed significantly upregulated p‐IκBα and p‐NF‐κB p65 expression in LPS‐stimulated BV‐2 cells and in the striatum of TD rats, suggesting an association between the IKK/NF‐κB pathway and TD development. Furthermore, JXZDF treatment suppressed the increased expression of proteins in this pathway in TD rats and BV‐2 cells, suggesting that its therapeutic effects occur through the modulation of this signaling cascade.

Microglia are the primary immune phagocytes in the CNS, and their overactivation can trigger neuroinflammatory responses, contributing to various psychiatric disorders (Shi and Yong 2025). Thus, inhibition of aberrant microglial activation is a promising area for investigating disease mechanisms and treatment (Guo et al. 2024). In the present study, JXZDF treatment alleviated LPS‐induced damage in microglia; however, an IKK inhibitor reversed this therapeutic effect, suggesting that microglial activation may be linked to the IKK/NF‐κB pathway, a finding that warrants further investigation.

This study has several limitations. Appropriate concentrations of JXZDF significantly inhibited TD symptoms; however, further investigations are needed to determine the optimal therapeutic concentration of JXZDF. Additionally, although JXZDF appears to exert its effect via modulation of the IKK/NF‐κB pathway, comprehensive experimental evidence, including in vivo and in vitro assessment of reversal using pathway‐specific inhibitors, is required to substantiate this hypothesis. Furthermore, specific IKK knockdown or overexpression in future studies may help establish a clearer causal relationship between JXZDF and the IKK/NF‐κB pathway.

## 5 Conclusions

JXZDF alleviates histopathological damage and modulates GABA and Glu expression in rats with TD. Moreover, the therapeutic efficacy of JXZDF during TD treatment may be mediated via regulation of the IKK/NF‐κB signaling pathway. These findings provide a theoretical foundation for the JXZDF use in TD treatment.

## Author Contributions

C. X. and F. H. led analysis and writing of the manuscript. F. F., J. Y. and Z. N. performed experimental operations. F. F. supervised the research. All authors read and approved the final manuscript.

## Funding

This work was supported by The Fundamental Research Funds for the Central Public Welfare Research Institutes (No. ZZ17‐XRZ‐043, 60102, 44002, 81330).

## Ethics Statement

All experimental and animal care procedures were approved by the Chinese Academy of Traditional Chinese Medicine Guang'anmen Hospital (approval No. IACUC‐GAMH‐2025‐019), and they conformed to the Guide for the Care and Use of Laboratory Animals produced by the National Institutes of Health and Animal Research: Reporting of In Vivo Experiments.

## Conflicts of Interest

The authors declare no conflicts of interest.

## Data Availability

The data sets used and analyzed during the current study are available from the corresponding author on reasonable request.
